# Identification of the Functional Domain of HPIV3 Matrix Protein Interacting with Nucleocapsid Protein

**DOI:** 10.1155/2020/2616172

**Published:** 2020-12-08

**Authors:** Xichuan Deng, Chaoliang Zhang, Kehan Zhang, Nan Lu, Yonglin He, Jia Liu, Zhibang Yang, Guangyuan Zhang

**Affiliations:** ^1^Pathogen Biology and Immunology Laboratory, Tissue and Cell Biology Laboratory, Experimental Teaching Management Center, Chongqing Medical University, Chongqing 401331, China; ^2^The First Clinical College of Chongqing Medical University, Chongqing 401331, China; ^3^Department of Pathogen Biology, Basic Medical College of Chongqing Medical University, Chongqing 401331, China

## Abstract

Human parainfluenza virus type 3 (HPIV3) is the main pathogen that causes respiratory infections in infants, young children, and the elderly. Currently, there are no vaccines and effective anti-infective drugs. Studying the replication and proliferation mechanism of HPIV3 is helpful for exploring the targets of anti-HPIV3 infection. Matrix protein (M) and nucleocapsid protein (N) are two key structural proteins of HPIV3 that exert important functions in HPIV3 proliferation. Herein, we aim to clarify the functional domains of M and N interaction. HPIV3 M and N expression plasmids of pCAGGS-HA-M and pCAGGS-N-Myc/Flag, M C-terminal truncation mutant plasmids of pCAGGSHA-M*Δ*C120, M*Δ*C170, M*Δ*C190, and M*Δ*C210, and M C-terminal plasmid of pCAGGS-HA-MC190 and C-terminal deletion mutant plasmid of pCAGGS-M*Δ*N143-182 were constructed. By using immunoprecipitation, immunofluorescence, and virus-like particle (VLP) germination experiments, we found that N was encapsulated into M-mediated VLP through N and M interaction. Moreover, the C-terminus of the M played a key role in the interaction between M and N. The C-terminus of the M encapsulated the N into the VLP. We finally determined that the 143-182 amino acids in the M were the functional regions that encapsulated the N into the M-mediated VLP. Our findings confirmed the interaction between M and N and for the first time clarified that the 143-182 amino acid region in M was the functional region that interacted with N, which provides a molecular basis for exploring effective anti-HPIV3 targets.

## 1. Introduction

Human parainfluenza virus (HPIV) belongs to paramyxovirus, which is a single- and negative-stranded and enveloped RNA virus. HPIV is a common pathogen of acute viral respiratory infections, which mainly causes upper respiratory tract and lower respiratory tract infections in children [1, 2]. In addition, it is also the main pathogen of respiratory tract infections in patients with immunodeficiency and chronic diseases, and the elderly [3, 4]. HPIV has 4 subtypes, namely, HPIV1~4. Among the 4 subtypes, HPIV3 is the most common and most pathogenic one. In infants and children, 30-40% of acute respiratory infections are caused by HPIV3 [5]. The pathogenicity of HPIV3 is second only to that of respiratory syncytial virus [6, 7]. A study has shown that lower respiratory tract infections caused by HPIV3 are one of the main causes of infant mortality [3]. However, there is currently no vaccine to prevent HPIV3 infection, and there is a lack of effective drugs against HPIV3 infection. Therefore, studying the replication and proliferation mechanism of HPIV3 and exploring the targets of anti-HPIV3 infection are of great clinical significance for the prevention and treatment of HPIV3 infection and the protection of human health, especially the health of infants and young children.

The HPIV3 genome is about 15 kb and encodes 6 main structural proteins, including matrix protein (M), nucleocapsid protein (N), phosphoprotein (P), RNA polymerase (L), hemagglutinin and neuraminidase (HN), and fusion protein (F) [8, 9]. HPIV3 replicates in the cytoplasm of infected cells, assembles on the plasma membrane, and then releases through germination to further infect other cells [10, 11]. In the replication process, the N wraps RNA to form an N-RNA template, which then forms an active complex RNP necessary for RNA transcription and replication together with L and cofactor P [12]. The M is composed of 353 amino acids and plays a key role in the assembly and germination of viruses [11, 13]. It is a nonintegrated, membrane-associated protein located under the lipid membrane of the virus and connected to the surface glycoprotein of the virus and RNP complex. However, the interaction between M and various components in the RNP complex is still unclear.

Studies have shown that the M of enveloped viruses is released outside the cell by germination and exists in the form of a virus-like particle (VLP). When the VLP germinates, it uses the lipid bilayer membrane of the host cell to encapsulate some viral structural proteins. VLP is very similar to the real virus in morphology and structure but does not contain the genome of the virus and is neither proliferative nor infectious [14, 15]. In addition to the formation of VLPs during the propagation of viruses in infected cells, VLPs can also be constructed artificially by expressing the necessary viral proteins in cell culture, laying the foundation for the development of VLP vaccines. VLP is also widely used to study virus assembly, germination mechanisms, and viral protein interactions. Similar to the M of other enveloped RNA viruses, expressing M of HPIV3 alone can form VLP [13, 16–20], while N cannot.

In this study, to clarify the functional domains of M and N interaction, we first constructed expression plasmids of HPIV3 M and N, namely, pCAGGS-HA-M, pCAGGS-N-Myc, and pCAGGS-N-Flag. The M was expressed in eukaryotic cells to form VLP and coexpressed with N. The interaction between N and M during the formation of HPIV3 VLP was analyzed by VLP germination experiment, coimmunoprecipitation, and immunofluorescence. In addition, by further constructing M truncation mutants and deletion mutants, the interaction domains of M with N during the formation of VLPs were identified. For the first time, a completely new functional domain of M interacting with N was elucidated, which provides a molecular basis for further research on the molecular mechanism underlying the interaction between N and M and the role of M in the assembly and germination of HPIV3 RNP complex.

## 2. Materials and Methods

### 2.1. Plasmid Construction

We designed and constructed the following plasmids: pCAGGS-N-Myc and pCAGGS-N-Flag, respectively, encoding the N fused with Myc and Flag tags at C-terminal; pCAGGS-HA-M, encoding the M fused to the HA tag at the N-terminal; pCAGGSHA-M*Δ*C120, M*Δ*C170, M*Δ*C190, and M*Δ*C210, respectively, encoding the C-terminal truncated mutant M with a deletion of 120, 170, 190, and 210 amino acids; pCAGGS-HA-MC120, MC170, and MC190, respectively, encoding M mutants with only C-terminal 120, 170, and 190 amino acids; and pCAGGS-HA-M*Δ*N143-182, encoding a mutant M in which the 143-182 amino acids were lacking. The plasmid pocus-HPIV3 containing the HPIV3 genome (gifted from Professor Mingzhou Chen, School of Life Sciences, Wuhan University) was used as a template for PCR amplification of the gene fragments of N or M mutants. The amplified fragments and plasmid pCAGGS-MCS were cut with EcoRI and NheI, respectively, and the digested fragments were cloned into pCAGGS-MCS. All constructed plasmids were verified by Sanger sequencing.

### 2.2. Cells and Cell Transfection

The 293T cells and HeLa cells (both from Key Laboratory of Infectious Diseases of Chongqing Medical University) were cultured with DMEM (HyClone) containing 16% fetal bovine serum (EveryGreen) and 1% penicillin-streptomycin (Gibco) and placed at 37°C, 5% CO_2_. When cell confluence reached 40%-50% for 293T cells and 50%-60% for HeLa cells, the cells were transfected with the plasmids with calcium phosphate reagent (Beyotime) for 293T cells and Lipo 3000 (Invitrogen) for HeLa cells.

### 2.3. Western Blot (WB)

At 48 h after transfection, 293T cells were collected and lysed with precooled TNE solution (50 mM Tris-Cl (pH 7.4), 150 mM NaCl, 2 mM EDTA (pH 8.0), 0.1% 2-mercaptoethanol, and protease inhibitor cocktail) for 30 min. After the lysate was centrifuged at 13,000 rpm, 4°C for 30 min, the supernatant was collected. Protein concentration was quantified by using the BCA Protein Assay Kit (Beyotime, Beijing, China). Then, protein electrophoresis was performed with 12% or 15% SDS-PAGE gel. The protein on the gel was transferred to a nitrocellulose membrane, and the membrane was blocked with 5% skimmed milk in PBST (1/1000 of Tween-20 dissolved in PBS) at room temperature for 30 min. Next, the corresponding primary antibody (HA, Sigma; Myc and GAPDH, Santa Cruz) diluted with PBST was added and incubated at room temperature for 1 h. After washing the membrane 3 times with PBST, the corresponding secondary antibody (HRP-conjugated goat anti-mouse IgG, Thermo Fisher) diluted with PBST was then incubated at room temperature for 45 min. After washing the membrane three more times, a developing substrate (HRP Substrate, Millipore Corporation) was added for color development.

### 2.4. VLP Germination and Quantification

The VLP germination experiment [21] was used to detect the amount of VLP germination in cell culture. Briefly, 48 h after transfection, 293T cells were collected and lysed as described above. The culture supernatant was collected, and cell debris was removed after centrifugation at 13,000 rpm for 1 min. Afterwards, the supernatant was slowly added to the 20% (wt/vol) sucrose solution and centrifuged at 40,000 rpm and 4°C for 2 h. After centrifugation, the VLP particles at the bottom of the tube were resuspended with 40 *μ*l TNE solution for WB analysis. The ability of M or its mutants to encapsulate N into VLP was defined as the germination index. In detail, germination index = the level of N in VLP/level of M or M mutant in VLP. The relative germination index was normalized to the germination index of M+N coexpression and was calculated as the ratio of (M mutants+N) group/(M+N) group.

### 2.5. Coimmunoprecipitation (Co-IP)

At 48 h after transfection, 293T cells were collected and lysed as described above. The lysates were incubated with anti-Myc (Santa Cruz) or anti-HA (Sigma) antibodies at 4°C for 1 h. After brief centrifugation, the samples were incubated with 40 *μ*l protein (A+G) agarose beads (Beyotime) overnight at 4°C. The next day, the sample was centrifuged at 8000 rpm for 10 seconds and then washed 5 times. Then, the samples were subjected to WB analysis.

### 2.6. Immunofluorescence

After 24 h of transfection in the HeLa cells, the cells were washed three times with PBS and then fixed with 4% paraformaldehyde at room temperature for 20 min. After washing three times, the cells were permeated with 0.2% Triton X-100 at room temperature for 20 min. After washing again, the cells were blocked with 3% bovine serum albumin solution for 30 min at room temperature. The N was stained with rabbit anti-Flag primary antibody (Proteintech) and AF488-conjugated goat anti-rabbit fluorescent secondary antibody (Thermo Fisher). The M was stained with mouse anti-HA primary antibody and AF568-conjugated goat anti-mouse fluorescent secondary antibody (Thermo Fisher). Finally, the cells were observed under a fluorescence microscope (Nikon Eclipse Ts2-FL, Japan).

### 2.7. Statistical Analysis

All experiments were performed at least three times. The data were shown as mean ± SD. The statistical analysis was performed using Student's *t*-test. A *p* value < 0.05 was considered as statically significant.

## 3. Results

### 3.1. M and N Interact on the Cytoplasm and Cell Membrane

**Figure 1:**
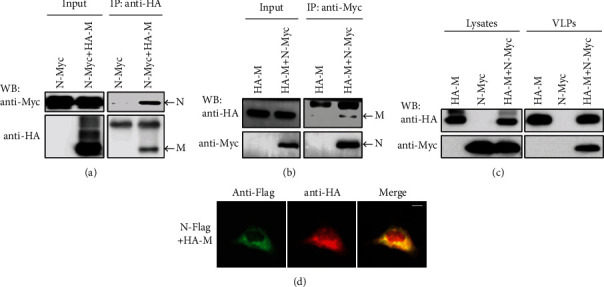
Interaction of N and M. (a, b) After 293T cells were transfected with the corresponding plasmids, the protein was immunoprecipitated with anti-HA (a) or anti-Myc (b). The protein level was detected by WB. (c) N-Myc and HA-M plasmids were used to transfect 293T cells alone or together, and the protein expression in cell lysate and VLP was detected by WB. (d) After cotransfecting HeLa cells with plasmids encoding N-Flag and HA-M, N was stained with anti-Flag and AF488-conjugated fluorescent secondary antibody, and M was stained with anti-HA and AF568-conjugated fluorescent secondary antibody. Immunofluorescence was observed under a microscope. Scale bar = 10 *μ*m.

In order to verify whether there is an interaction between M and N, Myc-labeled N and HA-labeled M were coexpressed in 293T cells, and the interaction between M and N was detected by Co-IP. It was found that Myc-N protein was coprecipitated with HA-M protein (Figure 1(a)). Similarly, HA-M protein was coprecipitated with Myc-N protein (Figure 1(b)). These results verify that there is indeed an interaction between M and N.

To explore the interaction between N and M in the germination of HPIV3 progeny virus, N and M were coexpressed in 293T cells, and M and N expressed alone were used as control, respectively. The results showed that N was detected in VLP of cells with coexpression of N and M, but not in VLP of cells with N or M expression alone (Figure 1(c)). These results suggest that N is encapsulated into M-mediated VLP through interaction with M. In addition, immunofluorescence results showed that both N and M had good colocalization in the cytoplasm and on the cell membrane (Figure 1(d)).

### 3.2. The C-Terminal of M Interacts with N

**Figure 2:**
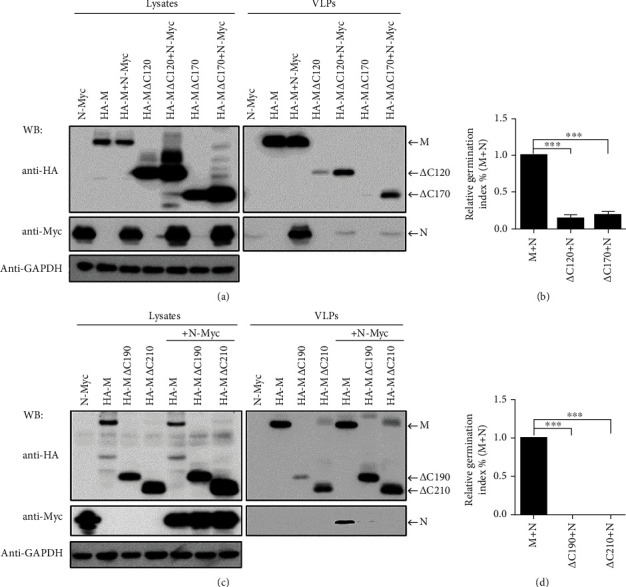
The C-terminal truncation mutant of M has a defect in its ability to encapsulate N into VLP. (a) N-Myc, HA-M, HA-M*Δ*C120, and HA-M*Δ*C170 plasmids were used to transfect 293T cells alone or together, and the proteins in cell lysate and VLP were detected by WB. (b) Relative germination index. The data is averaged from the results of three independent experiments. ^∗∗∗^*p* < 0.001. (c) N-Myc, HA-M, HA-M*Δ*C190, and HA-M*Δ*C210 plasmids were individually or cotransfected into 293T cells, and the proteins in cell lysate and VLP were detected by WB. (d) Relative germination index. The data is the average of the results of three independent experiments. ^∗∗∗^*p* < 0.001.

To further search for the functional domain where M interacts with N, we constructed plasmids expressing the C-terminal truncation mutants of M, including M*Δ*C120, M*Δ*C170, M*Δ*C190, and M*Δ*C210. Then, a VLP germination experiment was performed. In detail, compared with wild-type M, the ability of mutant M*Δ*C120 or M*Δ*C170 to encapsulate N into VLP was significantly reduced (Figures 2(a) and 2(b)). Compared with the germination index of M+N in VLP, the relative germination index of M*Δ*C120 and M*Δ*C170 was only 15-20% (Figure 2(b)). Although the mutants of M*Δ*C190 and M*Δ*C210 could produce an equivalent amount of VLP to that by wild-type M in the presence of N, N could not be detected in the VLP (Figure 2(c)). Moreover, compared to wild-type M, the mutants of M*Δ*C190 and M*Δ*C210 completely lost the ability to encapsulate N into VLP (Figure 2(d)).

**Figure 3:**
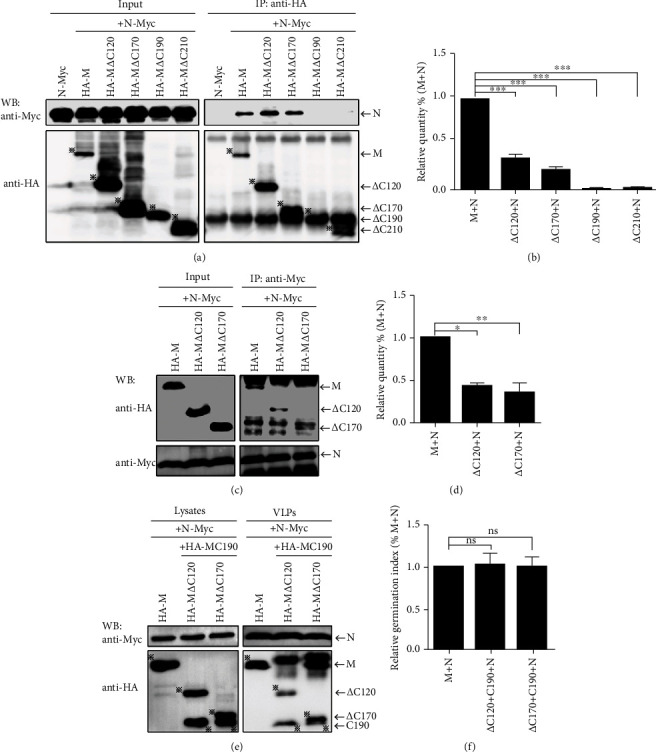
The C-terminal of the M alone can compensate for the ability of the C-terminal truncation mutant to encapsulate N into the VLP. (a) N-Myc, HA-M, M*Δ*C120, M*Δ*C170, M*Δ*C190, and M*Δ*C210 plasmids were transfected alone or cotransfected into 293T cells. After transfection, anti-HA was used for immunoprecipitation. The protein level was detected by WB. Asterisks indicate specific proteins detected. (b) The ability of M or mutant to interact with N. The data is from the average of the results of three independent experiments. ^∗∗∗^*p* < 0.001. (c) N-Myc plasmid was cotransfected with HA-M, M*Δ*C120, or M*Δ*C170 plasmids, respectively, into 293T cells, and after transfection, anti-anti-Myc was used for coimmunoprecipitation. The protein level was detected by WB. (d) Relative ability to interact with N in the same group as M+N and M*Δ*C120/M*Δ*C170+N. The data is derived from the average of the results from three independent experiments. ^∗^*p* < 0.05 and ^∗∗^*p* < 0.01. (e) N-Myc, HA-M, HA-MC190, HA-M*Δ*C120, or HA-M*Δ*C170 plasmids were cotransfected into 293T cells. After transfection, cells and VLPs were collected and protein expression was detected by WB. Asterisks indicate specific proteins detected. (f) Relative germination index of M and mutant integrating N into VLP. The data are from the average of three independent experiments. ns: not significant (*p* > 0.05).

Further Co-IP showed that although the levels of M*Δ*C120, M*Δ*C170, M*Δ*C190, and M*Δ*C210 proteins under IP were much greater than M, however, the level of N coprecipitated by M*Δ*C120 or M*Δ*C170 was only comparable to M (Figure 3(a)). Furthermore, the N did not interact with the two mutants M*Δ*C190 and M*Δ*C210 (Figure 3(a)). Therefore, compared with wild-type M, M*Δ*C120 or M*Δ*C170 protein had a serious defect in its ability to interact with N (Figure 3(b)), while M*Δ*C190 and M*Δ*C210 completely lose the ability to interact with N (Figure 3(b)). To further verify the results of the interaction between M*Δ*C120 and M*Δ*C170 with N, we conducted a reverse Co-IP experiment. The M*Δ*C120 or M*Δ*C170 coprecipitated by N was indeed much less than that of wild-type M (Figure 3(c)), indicating that the ability of M*Δ*C120 or M*Δ*C170 to interact with N is indeed significantly lower than that of M (Figure 3(d)). These results suggest that the C-terminal region of M plays an important role in the interaction with N.

### 3.3. C-Terminal Region of M Alone Encapsulates N into VLP through Interaction with N

We speculate that when the C-terminal region of M is coexpressed with C-terminal truncation mutants of M, the loss of interaction between the M C-terminal truncation mutant and the N can be partially restored. To verify this hypothesis, we constructed mutants that expressed the C-terminus of M, including MC120, MC170, and MC190. However, we failed to obtain the expression of the mutant proteins MC120 and MC170 (data not shown). Therefore, we only used MC190 for subsequent experiments. After cotransfection of plasmids pCAGGS-HA-MC190, pCAGGS-N-Myc, pCAGGS-HA-M*Δ*C120, M*Δ*C170, pCAGGS-HA-M, and pCAGGS-N-Myc, we detected the encapsulation of N into VLP. The results showed that when MC190 was present, the amount of M*Δ*C120 or M*Δ*C170 encapsulating N into VLP was equivalent to that of wild-type M (Figure 3(e)), which means that the ability to encapsulate N into VLP was comparable to M (Figure 3(f)). This result indicates that the C-terminal of M can compensate for the ability of the C-terminal truncated mutant to bind to the N.

**Figure 4:**
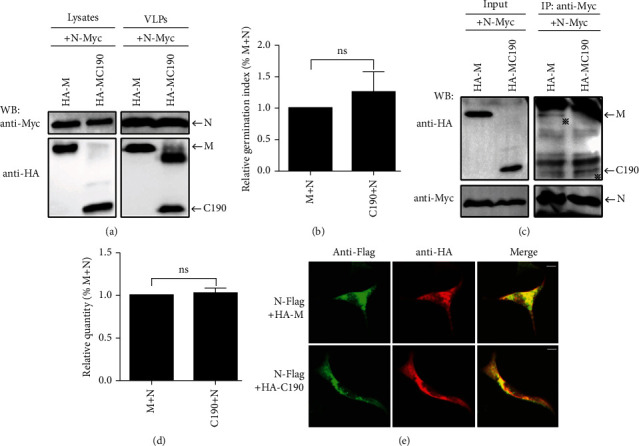
MC190 effectively encapsulates N into its VLP by interacting with N. (a) N-Myc plasmid was cotransfected into 293T cells with HA-M or HA-MC190 plasmids, respectively. After transfection, cells and VLPs were collected and protein expression was detected by WB. (b) The relative ability of M and MC190 to encapsulate N into VLP. The data are from the average of three independent experiments, ns: *p* > 0.05. (c) After transfection with N-Myc and HA-M or HA-MC190 plasmids, 293T cells were collected and immunoprecipitated with anti-Myc, and protein expression was detected by WB. Asterisks indicate specific proteins detected. (d) The relative ability of the M+N group or the MC190+N group to interact with N. The data are from the average of three independent experiments, ns: not significant (*p* > 0.05). (e) After transfection with N-Flag plasmid and HA-M or HA-MC190 plasmid, the HeLa cells were subjected to immunofluorescence analysis. Scale bar = 10 *μ*m.

To detect the interaction between MC190 and N, we coexpressed HA-MC190 and N-Myc and performed a VLP germination experiment. The results showed that although the content of MC190 in VLP was less than that of wild-type M, the ability of MC190 to encapsulate N into VLP was comparable to that of wild-type M (Figures 4(a) and 4(b)). The Co-IP results between MC190 and N showed that when a considerable amount of N-Myc was precipitated, the coprecipitated MC190 was also equivalent to M (Figures 4(c) and 4(d)), which was consistent with the VLP germination results. In addition, immunofluorescence analysis of MC190 and N localization showed that, like wild-type M, N could colocalize well with MC190 in cells (Figure 4(e)). The above results indicate that the C-terminus of M alone is sufficient to interact with N and encapsulate N into VLP.

### 3.4. The 143-182aa in M Is the Functional Domain That Encapsulates N into VLP

**Figure 5:**
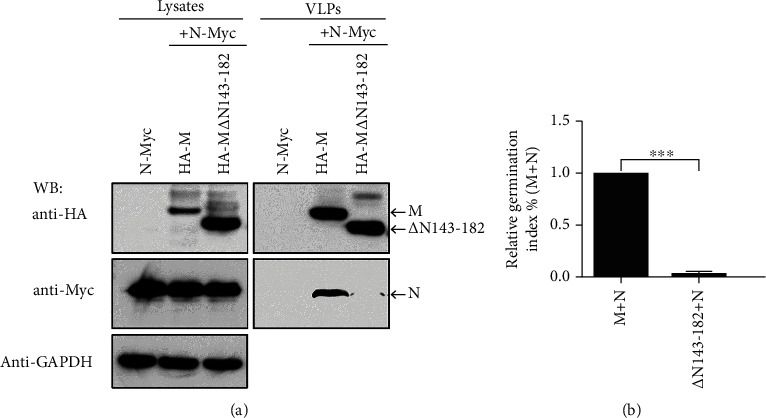
The 143-182aa in the M is the key region that encapsulates the N into the VLP. (a) N-Myc, HA-M, and M*Δ*N143-182 plasmids were transfected alone or cotransfected into 293T cells. After transfection, cells and VLPs were collected and protein expression was detected by WB. (b) The relative ability of M and M*Δ*N143-182 to encapsulate N into VLP. Data are from the average of three independent experiments, ^∗∗∗^*p* < 0.001.

As described above, M*Δ*C120 and M*Δ*C170 retained part of the ability to bind to N, while M*Δ*C190 and M*Δ*C210 had completely lost their ability to bind to N. Therefore, we speculate that M*Δ*C171-190 (i.e., 163-182 amino acids of M) or M*Δ*C171-210 (i.e., 143-182 amino acid regions of M) play an important role in N and M interactions. To verify this hypothesis, we cotransfected HA-M*Δ*N143-182 and N-Myc and tested VLP germination. As expected, although M*Δ*N143-182 mediated the production of a sufficient amount of VLP like wild-type M, it could not encapsulate the N into the VLP (Figure 5(a)). As in the case of the mutants M*Δ*C190 and M*Δ*C210, the ability of M*Δ*N143-182 to bind to the N was almost completely lost (Figure 5(b)). These results indicate that 143-182aa of the M is the functional domain that encapsulates the N into the VLP.

## 4. Discussion

M and N are two structural proteins encoded by the HPIV3 genome. Recent studies have shown that they have important functions in the proliferation of HPIV3 [22–24]. HPIV3 utilizes the inner membrane of the host cell to encapsulate RNP complexes and to form inclusion bodies, providing the necessary microenvironment for RNA transcription and replication [25]. M is the matrix protein of HPIV3 inner membrane, which plays a vital role in the assembly and germination of HPIV3, but its effect on virus replication is not yet clear. Studies have found that similar to other unsegmented negative-strand RNA viruses, HPIV3 M also regulates viral transcription and replication [17, 26, 27]. M reduces the formation of HPIV3 inclusion bodies in cells and the replication of RNA in inclusion bodies through interaction with N [23]. Therefore, exploring the functional domain of M and N interaction may reveal the molecular mechanism of viral transcription and replication, thus providing targets for developing anti-HPIV3 drugs.

In this study, we verified the interaction between the M and the N of HPIV3 through VLP germination experiment, immunoprecipitation, and immunofluorescence colocalization. We speculate that the interaction between N and M exists in the process of replication and assembly of HPIV3 progeny. Moreover, the interaction between N and M may start from the replication of the HPIV3 progeny virus, which exists during the assembly and germination of the progeny virus. The RNA and other structural proteins of the progeny virus may be assembled into a complete virus through the action of N and M. The complete viruses will be released from the cells as the M germinates.

We found that when the C-terminal truncated mutant of M was expressed alone, its ability to produce VLP was greatly lost compared to wild-type M, but when it was coexpressed with N, these mutants restored the ability to generate VLP in varying degrees. The recovery of the mutant VLP's germination ability is probably due to the increased expression level of N in the lysate, suggesting that the N may be able to increase the solubility of the M mutant in the lysate. However, although the VLP germination ability of the M truncation mutants was restored to some extent in the presence of N, their ability to encapsulate N into VLP was reduced compared to the wild-type M. Among them, the ability of M*Δ*C120 and M*Δ*C170 to encapsulate N into VLP was significantly reduced, while M*Δ*C190 and M*Δ*C210 completely lost the ability to encapsulate N into VLP. In addition, the C-terminal region of the M, namely, MC190, compensated for the ability of the C-terminal truncation mutant to bind to the N. One possible reason is that MC190 and the C-terminal truncation mutant are structurally and functionally complementary, performing functions equivalent to the wild-type M. Another possibility is that MC190 alone contains the functional domain where M interacts with N. We finally confirmed the latter speculation and further clarified that the key region of the M that interacted with the N was located at the C-terminus of the M.

A previous report indicates that the M L305 is a key residue, which plays an important role in the interaction of N and M, and the mutant L305A cannot interact with N nor can it encapsulate N into the VLP [28]. M consists of 353 amino acids, and L305 is located at the C-terminus. Therefore, the molecular mechanism of N-M interaction seems to be more complicated. It can be speculated that (i) there may be more than one region in the M that is responsible for the interaction with the N. In addition to the reported L305, we found that 143-182aa was another domain that also interacted with N. (ii) The above two known functional residues or regions may jointly promote the structural conformation of the M and its interaction with the N. Therefore, when any one of the two regions is defective, M cannot effectively interact with N.

In addition, in the N-M interaction, M and N have their own functional sites or regions involved. Although we have preliminarily determined the functional region in the M, the domain responsible for the N-M interaction in the N is still unknown. It has been reported that the C-terminus of paramyxovirus N may be involved in its interaction with P and M [17, 29]. Whether the C-terminal domain of HPIV3 N is a functional region that interacts with M still needs further verification. The M of RNA viruses, including influenza viruses and human respiratory syncytial viruses, can directly interact with RNA [30, 31]. Therefore, in the process of mediating the assembly of RNP, in addition to interacting with key proteins in the RNP complex, M may also directly interact with viral RNA, which needs to be further clarified by subsequent studies.

In this study, the new functional regions we identified in the M played a key role in the N-M interaction. These results provide a molecular basis for further revealing the interaction of virus proteins during virus proliferation and for identifying effective antiviral targets.

## Data Availability

The data used to support the findings of this study are available from the corresponding author upon reasonable request.
